# Gram-positive phage-host interactions

**DOI:** 10.3389/fmicb.2015.00061

**Published:** 2015-02-11

**Authors:** Jennifer Mahony, Douwe van Sinderen

**Affiliations:** ^1^School of Microbiology, University College CorkCork, Ireland; ^2^Alimentary Pharmabiotic Centre, University College CorkCork, Ireland

**Keywords:** interactions, lactic acid bacteria, phage attachment, host receptor, dairy, phage therapy

Bacteriophage research has seen many peaks and troughs over the past century ascending with phage therapy and application in the early 1900's; a research peak which was largely overshadowed by the dawning of the antibiotic era, and which has now deservedly regained attention as an approach against the problematic rise in antibiotic-resistant pathogenic bacteria. Following this initial scientific highlight, the advent of molecular biology and biotechnology sparked a renewed interest in phages and their encoded enzymes and promoters, which are still employed as research tools today. Much of this research was conducted using phages of Gram-negative bacteria, particularly *Escherichia coli*, due to the reliability of the host and the ease of protein (over) production, in particular many enzymes, in a compatible host background. Consequently, coliphages such as T4 and lambda served as model phages in the development of molecular tools and the fundamental understanding of phage-host interactions. The advent of new generation sequencing technologies has in recent years provided a vast array of sequence data relating to Gram-positive phages and their hosts, which in turn has permitted the development of analogies between Gram-negative and Gram-positive phages. For example, sequence analysis of *Bacillus subtilis* and *Lactococcus lactis* phages SPP1 and Tuc2009, respectively, revealed genomes with a conserved gene and/or functional order relative to lambda, the main model for *Siphoviridae* phages. While the Gram-negative models have been extremely useful platforms, many questions have remained unanswered owing to the fundamental structural and compositional differences between the cell walls of Gram-negative and positive cells. In response to this knowledge gap, there has been a significant upsurge in research in the area of phages infecting Gram-positive bacteria and in particular, lactococcal phage-host interactions, which have now become one of the leading model systems along with the above-mentioned *Bacillus subtilis* phage SPP1 and the mycobacteriophage L5.

In the ensuing 11 articles, many key advances that now define our understanding of phage-host interactions of Gram-positive bacteria and their infecting phages are described. We collate these advances and define the current knowledge of cell wall structures that present the target molecule of phage attachment (Munsch-Alatossava and Alatossava, 2013; Chapot-Chartier, 2014) and the phage-encoded adhesion complexes that phage employ to attach to their host in lactococci (Spinelli et al., 2014). Additionally, we explore the role of genomics in advancing knowledge on phages infecting previously underrepresented bacterial species that are of practical relevance to the food industry including the *Leuconostoc, Oenococcus* and *Weissella* (Kot et al., 2014; Mahony and van Sinderen, 2014), and phage therapy including *Listeria* and *Clostridium* spp. (Hagens and Loessner, 2014; Hargreaves and Clokie, 2014; Ly-Chatain, 2014). Furthermore, the research articles reinforce the continuing need for isolation and characterisation of phage isolates to retain a current perspective on the ever-changing phage genomics landscape (Cavanagh et al., 2014) and the possibility of deriving and understanding anti-phage measures that may be harnessed in various biotechnology sectors, in particular the dairy industry (Ali et al., 2014; Chirico et al., 2014).

## Conflict of interest statement

The authors declare that the research was conducted in the absence of any commercial or financial relationships that could be construed as a potential conflict of interest.
